# An Ultrasensitive Non-Enzymatic Sensor for Quantitation of Anti-Cancer Substance Chicoric Acid Based on Bimetallic Nanoalloy with Polyetherimide-Capped Reduced Graphene Oxide

**DOI:** 10.3390/nano10030499

**Published:** 2020-03-10

**Authors:** Jun Jiao, Meixin Pan, Xinran Liu, Jian Liu, Binshuai Li, Qiang Chen

**Affiliations:** The Key Laboratory of Bioactive Materials Ministry of Education, College of Life Science, Nankai University, Weijin Road No. 94, Tianjin 300071, China

**Keywords:** nanomaterials, bimetallic nanoalloy, non-enzymatic sensor, reduced graphene oxide, chicoric acid, anti-cancer

## Abstract

Exploiting effective therapies to fight tumor growth is an important part of modern cancer research. The anti-cancer activities of many plant-derived substances are well known, in part because the substances are often extensively distributed. Chicoric acid, a phenolic compound widely distributed in many plants, has drawn widespread attention in recent years because of its extraordinary anti-cancer activities. However, traditional methods for quantifying chicoric acid are inefficient and time-consuming. In this study, an ultrasensitive non-enzymatic sensor for the determination of chicoric acid was developed based on the use of an Au@Pt-polyetherimide-reduced graphene oxide (PEI-RGO) nanohybrid-modified glassy carbon electrode. Owing to the considerable conductivity of PEI-functionalized RGO and the efficient electrocatalytic activity of Au@Pt nanoalloys, the designed sensor exhibited a high capacity for chicoric acid measurement, with a low detection limit of 4.8 nM (signal-to-noise ratio of 3) and a broad linear range of four orders of magnitude. With the advantages provided by the synergistic effects of Au@Pt nanocomposites and PEI-RGO, the developed sensor also revealed exceptional electrochemical characteristics, including superior sensitivity, fast response, acceptable long-term stability, and favorable selectivity. This work provides a powerful new platform for the highly accurate measurement of chicoric acid quantities, facilitating further research into its potential as a cancer treatment.

## 1. Introduction

The disease burden of cancer continues to be a global issue. It is expected to be the most significant barrier to increased life expectancies worldwide. As reported by the World Health Organization (WHO) in 2018, cancer is estimated to affect 29.5 million people and will cause an estimated 16.4 million deaths in 2040 [1]. Even though various strategies, such as surgery, radiotherapy, and chemotherapy, are used to treat cancer, the survival rate of patients with malignant tumors remains low [2,3]. Therefore, it is imperative to exploit effective therapies against cancer progression. In recent years, various compounds extracted from plants have shown potential as therapeutics for treating diverse types of cancer [4,5].

Dietary compounds and nutrients give effective aid to humans for preventing diseases or inhibiting developments of disorder in patients [6,7]. Several natural compounds, such as resveratrol and berberine, have also been applied as anti-cancer drugs, as they are less toxic and have fewer side effects than some chemicals in clinical practice. Additionally, they are found abundantly in nature and are cost-effective to process [8]. Chicoric acid was isolated and identified as a phenolic compound in 1958 [9]. It is a derivative of caffeic acid and is widely distributed in numerous plants and vegetables, especially in *Echinacea* (a genus belonging to the Asteraceae family) [10]. It has received attention for its antioxidant abilities and as an anti-HIV and anti-inflammatory compound [11,12,13]. More importantly, chicoric acid is effective in preventing tumors from further developing; therefore, it has the potential to be used as an anticancer drug in the near future. For instance, chicoric acid has been shown to activate autophagy by elevating endoplasmic reticulum (ER) stress, which considerably slows the progression of gastric cancer [14]. In addition, chicoric acid can repress the activity of telomerase, leading to apoptosis in colon cancer cells [15]. Owing to these vital anti-cancer activities, a variety of methods have been developed to accurately measure chicoric acid quantities. The quantitation of chicoric acid can be achieved through several techniques, including planar chromatography, UV/visible spectra, high-performance liquid chromatography (HPLC), and Nuclear Magnetic Resonance (NMR) spectroscopy [16,17,18,19]. Although such analytic approaches are precise and efficient, they still suffer from intrinsic shortcomings like the long time required for operation. They also depend on sophisticated instruments and expensive reagents, as well as specially trained personnel. Furthermore, the real-time study of the pharmacodynamics of chicoric acid in living cells or tissues presents difficulties when using these methods. Therefore, to expedite the exploration of chicoric acid in the prevention and treatment of cancer, rapid, low-cost, and ultrasensitive techniques to detect chicoric acid are required.

Electrochemical sensing has great potential as a powerful platform for the quantitation of chicoric acid as it offers high sensitivity, rapid response, and practicality. High-performance sensors have been used widely to detect the presence of phenolic compounds and have provided satisfactory results [20,21]. Akhtar et al. proposed an electrochemical sensor for the determination of phenolic compounds with low detection limits, and the sensor succeeded in practical detection of river water samples [22]. Additionally, sensors based on ternary nanocomposites have been used to detect caffeic acid rapidly and accurately [23]. Importantly, electrochemical sensors have high biocompatibility, which enables them to study the target substances in living cells [24,25,26]. In this regard, electrochemical sensors have the potential to facilitate research into pharmacodynamic effects of chicoric acid at the cellular level. The performance of a sensor depends not only on the recognition elements, but also on various nanomaterials. Bimetallic nanomaterials have attracted greater attention than their noble counterparts because of the outstanding catalytic and electronic properties in construction of the sensing surface [27,28,29]. After the introduction of bimetallic nanoalloys, the consumption of expensive noble metals decreased and catalytic performance improved significantly [30]. Pt and Au nanoalloys have the capacity to improve electron transfer when modifying biosensors, making them ideal for the detection of chicoric acid [31,32,33,34]. Reduced graphene oxide (RGO) can be an acceptable platform for electrochemical sensing because it can enhance electrical conductivity, provide sufficient binding sites, and improve stability under various conditions [35]. However, the tendency of RGO to be irreversibly stacked and its dispersibility cannot be ignored, given that these drawbacks cause a serious decrease in the catalytic activity sites of RGO [36]. Polyetherimide (PEI), as a polymer with an abundance of amino-groups, was introduced to solve the dispersion issue and further enhance the electrical transfer [37].

**Scheme 1:**
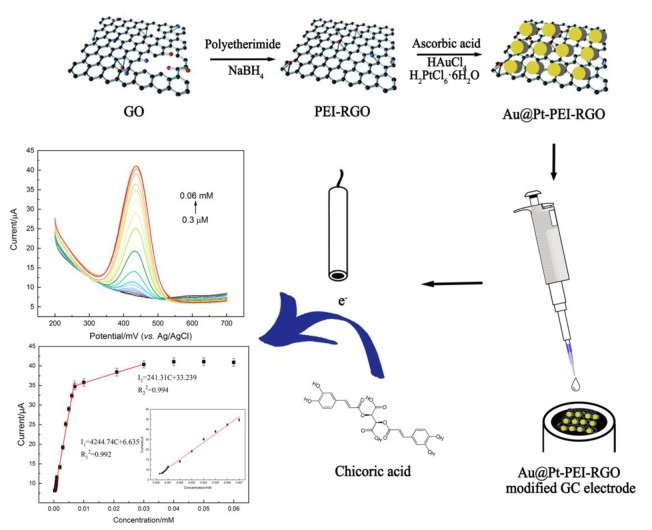
Schematic illustration for the preparation of Au@Pt-polyetherimide (PEI)-reduced graphene oxide (RGO)-modified glassy carbon electrode (GCE) applied for the detection of chicoric acid.

Herein, we explored a novel sensor for the determination of chicoric acid by modifying Au@Pt/PEI-RGO-layered nanohybrids on a glassy carbon electrode (GCE) (Scheme 1). To the best of our knowledge, this is the first report describing an electrochemical method for chicoric acid analysis. By combining the advantages of both bimetallic nanoalloys and PEI-capped RGO, our proposed sensor exhibited excellent responses to chicoric acid and obtained a detection limit as low as 4.8 nM, with a wide range spanning from 0.3 μM to 0.03 mM. This promising sensing platform for detecting chicoric acid, with high sensitivity and selectivity, paves the way for in-depth studies into the potential applicability of chicoric acid in the development of cancer therapy.

## 2. Materials and Methods

### 2.1. Reagents and Apparatus

Graphene oxide (GO) was acquired from Nanjing XFNANO Materials Tech Co. (Nanjing, China). Chicoric acid, gold chloride hydrate, platinic chloride hydrate, sodium borohydride (NaBH_4_), polyethyleneimine (PEI), ascorbic acid (AA), and hexadecyltrimethylammonium bromide (CTAB) were purchased from Sigma Aldrich Co. (St. Louis, MO, USA). Potassium chloride, ethanol, potassium ferricyanide, Na_2_HPO_4_, and citric acid were purchased from Tianjin Damao Chemical Reagent Factory (Tianjin, China). All the chemicals and reagents were of analytical reagent grade and used as received without further purification. Deionized water was used throughout the experiments unless otherwise indicated.

A conventional one-compartment cell using a 283 potentiostat–galvanostat electrochemical workstation (EG & G PARC using the M270 software) was used for the electrochemical experiments with highly pure nitrogen bubbled through the experimental solution for 20 min. A Pt wire (1 mm diameter) was used as the counter electrode, an Ag/AgCl electrode (saturated with KCl) was used as the reference electrode, and the Au@Pt-PEI-RGO-modified GC electrode was used as the working electrode (3 mm diameter); these comprised the conventional three-electrode system.

Transmission electron microscopy (TEM) and energy-dispersive X-ray spectroscopy (EDX) patterns were gathered on a Tecnai G2 F20 instrument (Philips Holland). X-ray diffraction (XRD) images were collected by a Rigaku D/max-rA with Cu Kα radiation (λ = 1.5418 Å) (Rigaku, Japan). A Raman spectrometer (model Renishaw in Via, Renishaw, England) was used to collect the Raman spectra.

### 2.2. Preparation of PEI-Functionalized RGO

GO (40 mg) was dispersed in 40 mL double distilled water by ultrasonication. After 2 h ultrasonication, polyethylene imine (PEI) was added to the reaction solution, followed by potassium hydroxide (KOH) until reaching pH 10.0, and then the solution was stirred for 2 h to achieve a homogeneous PEI-GO aqueous dispersion. The mixture reacted under magnetic stirring at 60 °C for 12 h and was centrifugated and dispersed in double distilled water to obtain PEI-RGO. According to the mass ratio of GO/NaBH4 = 1:11.4, 0.45 g NaBH4 was added into 10 mL PEI-RGO solution. After a 2 h reaction under magnetic stirring, the product was collected by centrifugation at 20,000 rpm for 30 min and washed three times with double distilled water. Finally, the product was resuspended to obtain 1 mg/mL PEI-RGO solution.

### 2.3. Preparation of Au@Pt-PEI-RGO

Hexadecyl trimethyl ammonium bromide (CTAB) solution (25 mL 0.1 M), 100 μL 1 M HAuCl_4_ solution, 100 μL 1 M H_2_PtCl_6_ solution, and 0.1 M ascorbic acid were mixed with the prepared PEI-RGO solution and reacted for 24 h with continuous stirring at room temperature. Finally, the products were obtained through centrifugation at 13,000 rpm and washed three times with double distilled water. The Au-PEI-RGO was prepared using a similar method.

### 2.4. Preparation of Modified Electrodes

The glassy carbon (GC) electrode was polished by 0.3 and 0.05 μm aluminum oxide powder and ultrasonically washed with double distilled water and absolute ethanol, respectively, followed by drying with a nitrogen stream. A 10 μL aliquot of the Au@Pt-PEI-RGO suspension was immobilized on the surface of the GC electrode by dip-coating and dried in air.

## 3. Results and Discussion

### 3.1. Characterization of Au@Pt-PEI-RGO Nanocomposites

**Figure 1:**
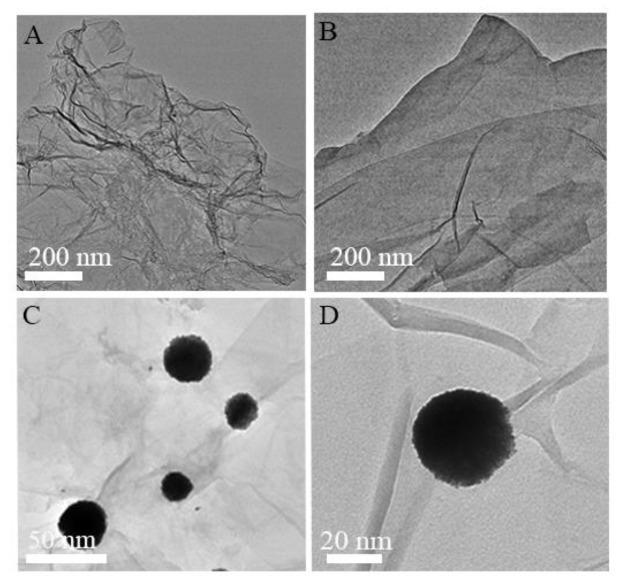
Transmission electron microscopy (TEM) images of (**A**) RGO, (**B**) PEI-functionalized RGO, and (**C**,**D**) Au@Pt-PEI-RGO under different magnification.

Transmission electron microscopy (TEM) was used to identify the morphological properties of nanoparticles prepared as described. A typical wrinkled pattern was observed in the RGO sheets (Figure 1A). After functionalization of PEI, RGO exhibited less aggregation than its original counterparts (Figure 1B). Under different magnification (Figure 1C,D), it was obvious that the Au@Pt alloy nanoparticles were well distributed on the PEI-RGO sheets and a single Au@Pt nanoparticle was approximately 30 nm in diameter.

**Figure 2:**
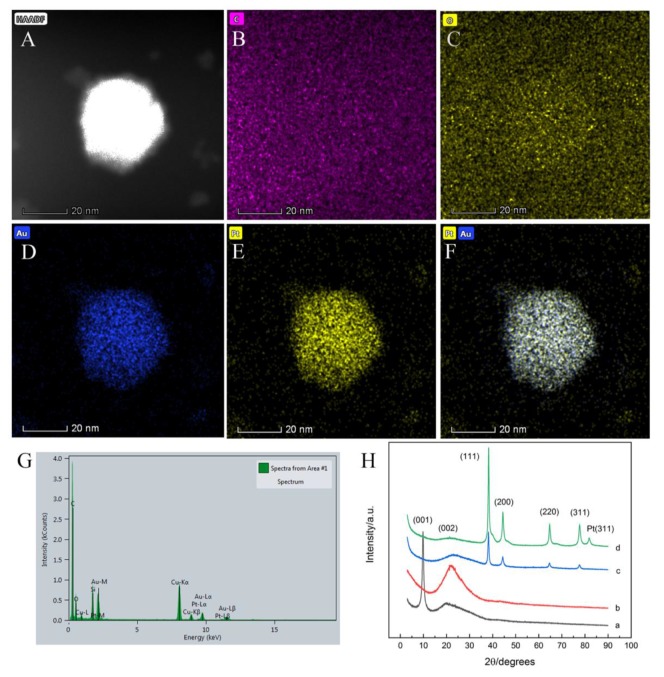
Images of (**A**) Au@Pt-PEI-RGO and elements of (**B**) C, (**C**) O, (**D**) Au, (**E**) Pt, and (**F**) Au@Pt; (**G**) energy-dispersive X-ray spectroscopy (EDX) spectrum of Au@Pt-PEI-RGO; (**H**) X-ray diffraction (XRD) patterns of (curve a) GO, (curve b) PEI-RGO, (curve c) Au-PEI-RGO, and (curve d) Au@Pt-PEI-RGO.

Elemental mapping, as a typical measurement, was used to display the structure and elemental distributions of the obtained nanocomposites. As shown in Figure 2A–F, it is clear that the C and O elements were equally distributed as a film, while Pt and Au were relatively concentrated, indicating that the nanoalloy was uniformly composed of Au and Pt and distributed throughout the surface of PEI-RGO. In addition, the occurrence of C, O, Au, and Pt elements in the energy-dispersive X-ray (EDX) spectrum (Figure 2G) further supports the formation of Au@Pt-PEI-RGO nanohybrids.

Figure 2H shows the X-ray diffraction (XRD) patterns of GO, PEI-RGO, Au-PEI-RGO, and Au@Pt-PEI-RGO. Compared with GO (curve a), with diffraction patterns at around 10° and 23.99°, RGO (curve b) displays a wide peak only at 23.99°, indicating that GO was reduced [38]. The patterns of Au-PEI-RGO (curve c) exhibit diffraction peaks of (111), (200), (220), and (311) at 38.2°, 44.3°, 64.7°, and 77.7°, respectively [39]. In contrast, for Au@Pt-PEI-RGO (curve d), a peak at 81.6° is associated with (311) facets of Pt nanoparticles, and other diffraction patterns are broader owing to the superposition of lattice planes, suggesting the formation of a bimetallic nanoalloy [40]. In addition, the Raman spectra in Figure S1 also reflect the structural changes that occurred during the formation of PEI-RGO. Compared with the Raman spectrum of GO, the increased D/G intensity ratio increased from 0.91 (GO) to 1.02 (PEI-RGO), suggesting a decline in the average size of the in-plane sp^2^ domains upon reduction of GO [41,42].

### 3.2. Electrochemical Behavior of Obtained Materials

**Figure 3:**
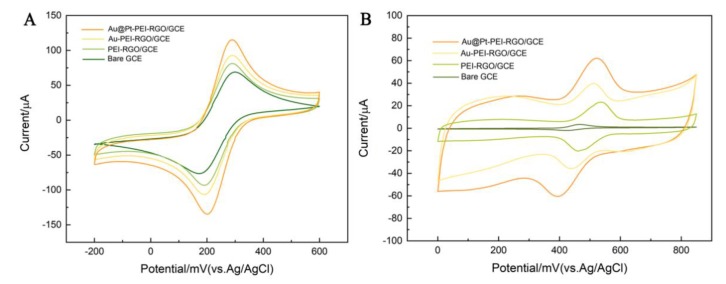
(**A**) Cyclic voltammograms (CVs) of bare GCE (green), PEI-RGO/GC electrode (light green), Au-PEI-RGO/GC electrode (yellow), and Au@Pt-PEI-RGO/GC electrode (orange) recorded in 0.1 M KCl solution containing 10 mM [Fe(CN)_6_]^3−^. Scan rate: 50 mV/s. (**B**) CVs of bare GCE (green), PEI-RGO/GC electrode (light green), Au-PEI-RGO/GC electrode (yellow), and Au@Pt-PEI-RGO/GC electrode (orange) in Na_2_HPO_4_-citric acid solution (pH 3) containing 0.5 mM chicoric acid.

The electrochemical performance of bare GCE, PEI-RGO/GCE, Au-PEI-RGO/GCE, and Au@Pt-PEI-RGO/GCE was assessed in cyclic voltammograms (CV) using ferro/ferricyanide as the redox probe. As shown in Figure 3A, compared with bare GCE (green), the electrode modified with PEI-RGO (light green) displayed a rising response to the redox probe, as the PEI-RGO accelerated the electron transport between ferrocyanide and electrode [43]. The introduction of bimetallic nanomaterials followed by PEI-RGO caused an elevated current response compared with Au-PEI-RGO-modified GCE (yellow), which can be ascribed to the synergistic effects between Au and Pt. To further discriminate the obtained materials, the active surface area was evaluated according to the Randles–Sevcik Equation [44]:

(1) $$Ip=2.69\times 10^{5}\times A\times D^{1/2}\times n^{3/2}\times v^{1/2}\times c$$

where *I_p_* represents the redox peak current; *A* is the electroactive surface area (cm^2^); the diffusion coefficient (*D*) of the molecule in solution is (6.70 ± 0.02) × 10^−6^ cm^2^/s; *n* relates to the number of electrons participating in the redox reaction, which is equal to 1; *v* corresponds to the scan rate (V/s); and *c* is the concentration of the redox probe (mol/cm^3^). The calculated electroactive area of Au@Pt-PEI-RGO-modified GCE was 0.086 cm^2^, which was 1.84 times, 1.46 times, and 1.26 times higher than bare GCE, PEI-RGO/GCE, and Au-PEI-RGO/GCE, respectively. Furthermore, the peak potential separation (ΔEp) of bare GCE was found to be around 124 mV. Whereas, we observed that Au@Pt-PEO-RGO modified GCE had a lower peak potential separation (ΔEp) of 88 mV, indicating that the obtained nanocomposites facilitated a faster electron transfer towards the electrode in bulk solution. In addition, the ratio of peak current (Ipa/Ipc) of Au@Pt-PEI-RGO/GCE was calculated to be ~1.005, which means the oxidation/reduction process of the Fe^2+^/Fe^3+^ species takes place reversibly [45]. The above results demonstrate that Au@Pt-PEI-RGO nanocomposites can enhance the electroactive area of the electrode, which facilitates electron transfer between the working electrode and K_3_[Fe(CN)_6_]. The excellent performance may be attributed to not only the enlarged supporting area provided by PEI-functionalized RGO with plenty of binding sites, but also the outstanding electrical conductivity and the synergistic effects from the bimetallic nanoalloy. Therefore, Au@Pt-PEI-RGO was proven to be an excellent electron transfer mediator and is applicable for electrochemical analysis.

### 3.3. Electrochemical Response of Chicoric Acid at Au@Pt-PEI-RGO/GCE

Figure 3B presents the electrocatalytic performance of the electrodes modified with different materials in detecting chicoric acid in an Na_2_HPO_4_-citric acid solution (pH 3) containing 0.5 mM chicoric acid at a scan rate of 50 mV/s. Only a small current response occurred on the bare GCE, whereas the modified electrodes all attained a couple of well-defined redox peaks. Compared with PEI-RGO/GCE and Au-PEI-RGO/GCE, a stronger response was observed at around 0.4 V/0.52 V on the electrode with bimetallic nanoalloys and PEI-RGO sheets, indicating that Au@Pt-PEI-RGO nanohybrids possess the strongest electrocatalytic properties for the oxidation reaction of chicoric acid. In addition, the ratio of peak current (Ipa/Ipc) of Au@Pt-PEI-RGO/GCE was around −1.025, which refers to a reversible process on the electrode. The value of Ipa/Ipc is close to 1, suggesting that the electrode displays a significantly catalytic performance [46]. The improvement in response signals may be attributed to the high electrocatalytic activity of gold and platinum, the electrical conductivity and enlarged active surface area of PEI-RGO, and the synergistic effects after integration.

### 3.4. Performance of the Proposed Sensor

#### 3.4.1. Optimization of the Experimental Variables

**Figure 4:**
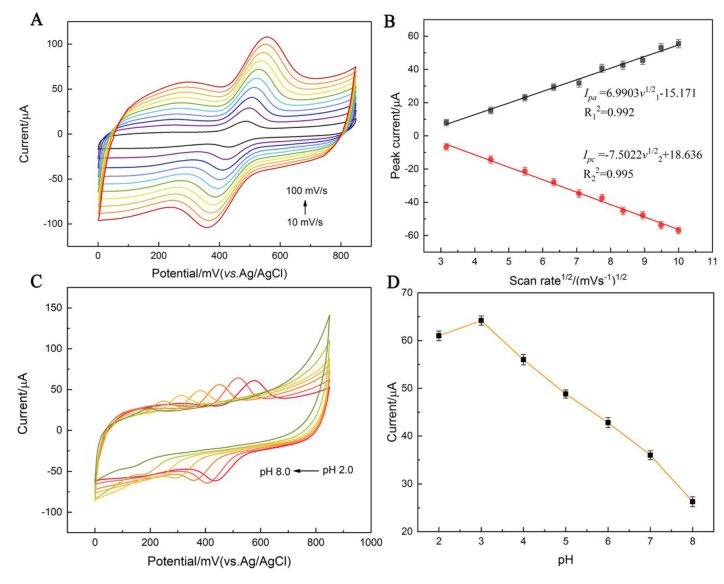
(**A**) CVs of the Au@Pt-PEI-RGO/GCE toward 0.5 mM chicoric acid in Na_2_HPO_4_-citric acid solution (pH 3) with different scanning rates (10, 20, 30, 40, 50, 60,70, 80, 90, and 100 mV s^−1^). (**B**) Linear relationship of the redox peak current (I_p_) versus the square root of scan rate (v^1/2^). Error bars = ± standard deviation, *n* = 5. (**C**) CVs responses to 0.5 mM chicoric acid in different pH conditions of Na_2_HPO_4_-citric acid solution (pH 2, 3, 4, 5, 6, 7, and 8) with Au@Pt-PEI-RGO/GCE. (**D**) The linear relationship of the oxidation potential versus the pH. Error bars = ± standard deviation, *n* = 5.

To obtain the best analytical performance from the proposed sensor, the effects of scan rate and solution pH on the redox of 0.5 mM chicoric acid were investigated. As shown in Figure 4A,B, the redox peak currents of chicoric acid are proportionate to the square root of the scan rate (v^1/2^) in the range of 10–100 mV/s, and the linear regression equations generated were I_pa_ = 6.9903v^1/2^ − 15.171 (R^2^ = 0.992) and I_pc_ = −7.5022v^1/2^ + 18.636 (R^2^ = 0.995). The above results indicate that the redox reaction of chicoric acid on the Au@Pt-PEI-RGO/GCE is predominantly a diffusion-controlled process [47,48]. In addition, the scanning rate was chosen as 50 mV/s to ensure the stability of subsequent electrocatalytic measurements.

The influence of pH value on the electrochemical performance of Au@Pt-PEI-RGO/GCE was tested by CV in an Na_2_HPO_4_-citric acid buffer containing 0.5 mM chicoric acid with pH values from 2 to 8. As seen in Figure 4C, the peak current and peak potential of the response curves were affected by the pH value. The redox peaks of chicoric acid at Au@Pt-PEI-RGO/GCE increased incrementally until reaching pH 3, whereas beyond pH 3, the current decreased with the consistent increase in pH (Figure 4D). Therefore, to accomplish the sensitive detection of chicoric acid, an Na_2_HPO_4_-citric acid buffer solution with pH 3 was selected as the optimal bulk electrolyte for further experiments.

#### 3.4.2. Electrochemical Determination of Chicoric Acid

**Figure 5:**
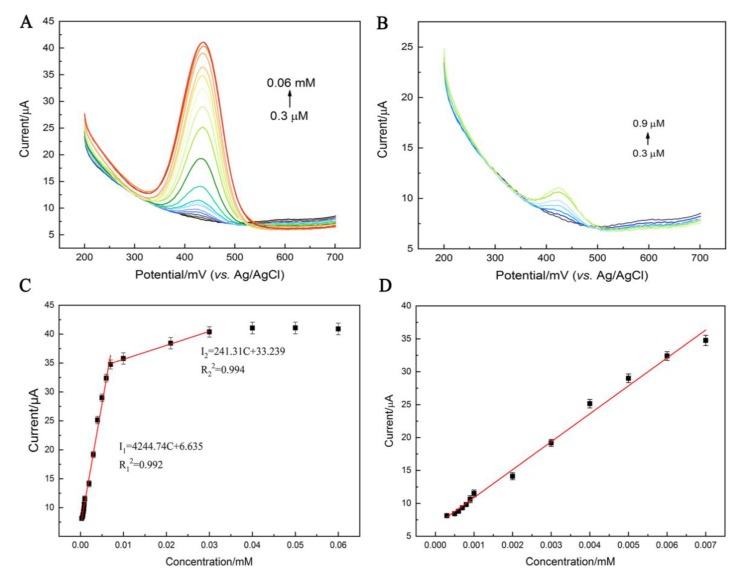
(**A**) Differential pulse voltammetry (DPV) of Au@Pt-PEI-RGO/GCE obtained in Na_2_HPO_4_-citric acid buffer solution (pH 3) containing various concentrations of chicoric acid. (**B**) A close-up of the low concentration of the chicoric acid region from 0.3 μM to 0.9 μM. (**C**,**D**) The corresponding calibration curve of response current versus the concentration of chicoric acid.

Differential pulse voltammetry (DPV) was performed to examine the relationship between the response currents and the concentration of chicoric acid at the Au@Pt-PEI-RGO-modified GCE under optimal conditions. Figure 5A illustrates the DPV profiles of different concentrations of chicoric acid from 0.3 μM to 0.06 mM, and Figure 5B presents a close-up of the low concentrations from 0.3 μM to 0.9 μM. As the concentration of chicoric acid increased, the oxidation peak current gradually rose. We fitted the calibration curve after five independent tests. From the calibrated current–concentration profile, the Au@Pt-PEI-RGO/GCE sensor showed two linear ranges of 0.3 μM to 0.007 mM and 0.007 mM to 0.3 mM with regression coefficient (R^2^) value of 0.992 and 0.994, respectively. The corresponding linear regressions can be represented by equations I_1_ = 4244.74C + 6.635 and I_2_ = 241.31C + 33.239. On the basis of three times the blank standard deviation and the slope of the calibration curve, the theoretical limit of detection was calculated as 0.0048 μM (S/N = 3), with a sensitivity of 49.357 μA μM^−1^·cm^−2^ [49].

Table 1 compares the analytical performance of our proposed sensor with that of other reported methods of chicoric acid determination. The Au@Pt-PEI-RGO nanocomposites-based sensor offers a wider linear range, simpler operation, and much lower detection limit than the other methods, providing a powerful tool for the development of chicoric acid-related cancer drugs. The above results reveal that the obtained Au@Pt-PEI-RGO nanohybrid is an attractive electrode modifier for the ultrasensitive determination of chicoric acid because of the enhanced electric conductivity provided by PEI-RGO, highly catalytic nature of Au@Pt nanoalloys, and their possible synergistic effects.

#### 3.4.3. Interference Immunity, Repeatability, and Stability Studies

**Figure 6:**
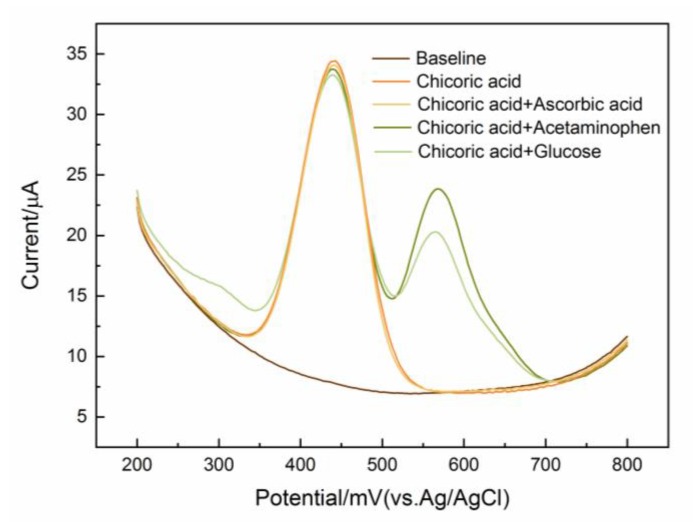
DPV responses of Au@Pt-PEI-RGO/GCE for 0.01 mM chicoric acid in the presence of an excess concentration of interfering substances.

Anti-interference capacity, repeatability, and long-term stability are significant parameters for evaluating the accuracy of the proposed sensor. Figure 6 displays the selectivity assessed by DPV in the presence of a 25-fold excess of relevant interfering substances, including ascorbic acid, acetaminophen, and glucose. No significant shift in the oxidation peak potential and an imperceptible change in response signals were seen in the presence of interfering substances, suggesting that the designed sensor based on Au@Pt-PEI-RGO exhibits a distinct specificity for chicoric acid. To explore the repeatability, parallel DPVs were implemented for the detection of chicoric acid on five independent Au@Pt-PEI-RGO/GCE. As shown in Figure S2, the relative standard deviation (RSD) was calculated to be 2.54%, suggesting favorable repeatability. In addition, the stability of the Au@Pt-PEI-RGO-modified electrode was also evaluated. After 30 days of storage, the proposed sensor was stable and retained 95.56% of the initial current response of the as-prepared sensor, indicating adequate long-term stability. Thus, the Au@Pt-PEI-RGO nanocomposite enjoys a satisfactory capacity for selectivity, repeatability, and long-term stability, facilitating the construction of a highly sensitive chicoric acid sensor.

## 4. Conclusions

In conclusion, we designed an ultrasensitive non-enzymatic sensor for the determination of chicoric acid based on Au@Pt-PEI-RGO nanocomposites with a facile synthesis. Adopting PEI-functionalized RGO as a substrate compellingly enhanced electron transfer and increased the electroactive surface area. Additionally, Au@Pt nanoalloys exhibited an exceptional electrocatalytic capacity for the oxidation of chicoric acid, promoting the electrochemical performance of the as-prepared electrode. The obtained sensor demonstrated effective chicoric acid detection in the concentration range of 0.3 μM to 0.03 mM, with a sensitivity of 49.357 μA μM^−1^·cm^−2^. A detection limit as low as 4.8 nm was achieved, which is a marked improvement over its traditional counterparts. This work paves the way for the more precise detection and quantitation of chicoric acid and provides a powerful platform to enhance research into the use of chicoric acid in cancer therapy.

## Figures and Tables

**Table 1:** Comparison of the reported methods for chicoric acid determination. PEI, polyetherimide; RGO, reduced graphene oxide.

Methods	Detection Range	Limit of Detection	Reference
High-performance liquid chromatography (HPLC)	0.25 M–1.25 M	11 mM	[50]
Liquid chromatography coupled with tandem mass spectrometry (HPLC–MS/MS)	0.05 mM–2 mM	2.23 μM	[51]
Capillary electrophoresis (CE)	74.1 mM–592.8 mM	1.1 mM	[52]
Electrochemical sensor based on Au@Pt-PEI-RGO nanohybrids	0.3 μM–0.03 mM	4.8 nM	This work

## Supplementary Materials

The following are available online at https://www.mdpi.com/2079-4991/10/3/499/s1, Figure S1: The Raman spectra of GO and PEI-RGO. Figure S2: Column graph of PDV signals in Na_2_HPO_4_-citric acid buffer solution (pH 3) containing 0.01 mM of chicoric acid at five different electrodes prepared under the same conditions.
