# Supplementary material for: An Ultrasensitive Non-Enzymatic Sensor for Quantitation of Anti-Cancer Substance Chicoric Acid Based on Bimetallic Nanoalloy with Polyetherimide-Capped Reduced Graphene Oxide
Source: Nanomaterials (Basel). 2020 Mar 10;10(3):499. doi: 10.3390/nano10030499 (PMC7153584; doi:10.3390/nano10030499)
Supplement: Supplementary file 1 [file nanomaterials-10-00499-s001.pdf]

Supporting information

An ultrasensitive non-enzymatic sensor for quantitation of anti-cancer substance chicoric acid based on bimetallic nanoalloy with polyetherimide-capped reduced graphene oxide

Jun Jiao, Meixin Pan, Xinran Liu, Jian Liu, Binshuai Li, Qiang Chen *

The Key Laboratory of Bioactive Materials Ministry of Education, College of Life Science, Nankai University, Weijin Road No. 94, Tianjin 300071, PR China

---

*Corresponding author:

E-mail address: qiangchen@nankai.edu.cn (Q. Chen).

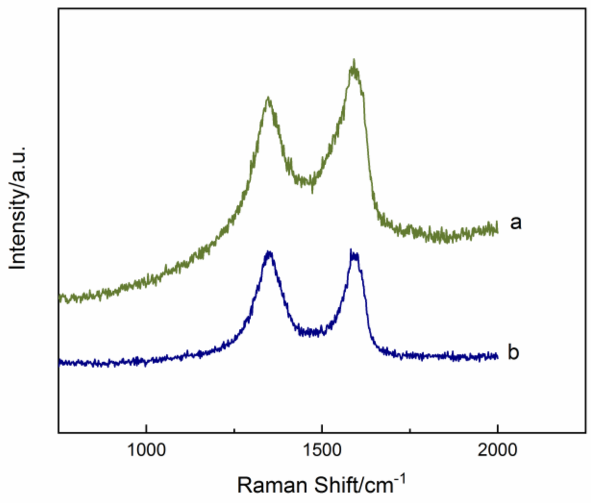

Figure S1.The Raman spectra of GO(a) and the PEI-RGO(b).

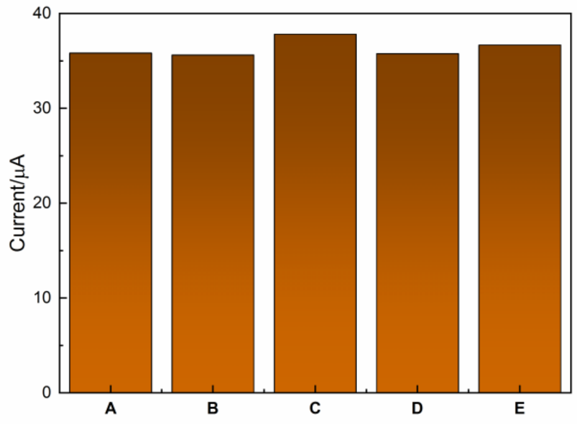

Figure S2. Column graph of PDV signals in Na_2HPO_4-citric acid buffer solution (pH 3) containing 0.01mM of chicoric acid at five different electrodes prepared under the same conditions.
